# Increased Nuclear Transporter Importin 7 Contributes to the Tumor Growth and Correlates With CD8 T Cell Infiltration in Cervical Cancer

**DOI:** 10.3389/fcell.2021.732786

**Published:** 2021-09-28

**Authors:** Jing Chen, Yan Hu, Yincheng Teng, BiKang Yang

**Affiliations:** ^1^Department of Obstetrics and Gynecology, Shanghai Jiao Tong University Affiliated Sixth People’s Hospital, Shanghai Jiao Tong University School of Medicine, Shanghai, China; ^2^Department of Gynecologic Oncology, Hunan Cancer Hospital, The Affiliated Cancer Hospital of Xiangya School of Medicine, Central South University, Changsha, China

**Keywords:** IPO7, mass spectrometry, proteome, immune infiltration, cervical cancer

## Abstract

**Background:** Importin 7 (IPO7), a karyopherin-β protein, is involved in various tumorigenesis and progression abilities by mediating the nuclear import of oncoproteins. However, the exact biological functions of IPO7 remain to be further elucidated.

**Materials and Methods:** TCGA and GEO datasets were used to identify dysregulated expression of IPO7 in various cancers. Gain-of-function and loss-of-function analyses were used to identify the oncogenic functions of IPO7 *in vitro* and *in vivo*. Moreover, LC-MS/MS and parallel reaction monitoring analysis were used to comparatively profiled IPO7-related proteomics and potential molecular machinery.

**Results:** Our works demonstrated that the expression of IPO7 was upregulated and was correlated with a poor prognosis in cervical cancer. *In vitro* and *in vivo* experiments demonstrated that knockdown of IPO7 inhibited the proliferation of HeLa and C-4 I cells. LC-MS/MS analysis showed that IPO7-related cargo proteins mainly were enriched in gene transcription regulation. Then independent PRM analysis for the first time demonstrated that 32 novel IPO7 cargo proteins, such as GTF2I, RORC1, PSPC1, and RBM25. Moreover, IPO7 contributed to activating the PI3K/AKT-mTOR pathway by mediating the nuclear import of GTF2I in cervical cancer cells. Intriguingly, we found that the IPO7 expression was negatively correlated with CD8 T cell infiltration *via* regulating the expression of CD276 in cervical cancer.

**Conclusion:** This study enhances our understanding of IPO7 nuclear-cytoplasmic translocation and might reveal novel potential therapeutic targets. The results of a negative correlation between the IPO7 and CD8 T cell infiltration indicate that the IPO7 might play an important impact on the immune microenvironment of cervical cancer.

## Introduction

Cervical cancer (CC) is the fourth most common cancer and has become a major challenge to female health (Sung et al., 2021). Although an increasing number of studies have been performed on CC, the treatment options and effects of advanced-stage CC remain limited (Fontham et al., 2020). Therefore, deeper comprehension of the potential biological molecular mechanisms of CC tumorigenesis and progression and the authentication of specific tumor markers is of enormous importance.

Nuclear-cytoplasmic translocation of macromolecule proteins (>40 KD) is an active process and is mediated by classical transport receptors, which include importins and exportins (Gorlich et al., 1997). Karyopherins delicately coordinate the spatial distribution of macromolecule molecules, including transcription factors and nuclear proteins, through a specialized nuclear pore complex (NPC; Sun et al., 2016). Accumulating evidence suggests that karyopherins directly interact with a variety of tumor-associated proteins and play crucial roles in cell cycle regulation, transcriptional regulation, cell apoptosis, and DNA repair (Cagatay and Chook, 2018). Dysregulation of nuclear-cytoplasmic translocation is an important cause of abnormal tumor-related proteins localization, which is closely related to cell proliferation, apoptosis, and immune response (Li et al., 2018; Du et al., 2020). For example, overexpression of KPNA2 participated in tumor immune evasion *via* regulating PD-L1 expression in pancreatic ductal adenocarcinoma (Zhou et al., 2021). Overexpression of karyopherin is associated with poor prognosis in multiple tumors, and targeting these key transporters represents a novel strategy for tumor treatment (Mahipal and Malafa, 2016).

Importin 7 (IPO7) protein is a member of the karyopherin-β protein family that participates in the trafficking of numerous proteins from the cytoplasm (Zaitseva et al., 2009). IPO7 has been demonstrated to directly interact with nuclear import signals (NLSs) of the ribosomal protein RPL23A and mediate its nuclear import (Golomb et al., 2012). On the other hand, IPO7 also contributes to the nuclear import of supercoiled plasmid DNA and SMAD3 in an NLS-independent manner (Chuderland et al., 2008; Dhanoya et al., 2013). Karyopherins have been well documented as pivotal receptors of nuclear-cytoplasmic transport and contribute to tumor progression mainly by transporting cargo proteins. For example, KPNA2 is involved in the regulation of DNA damage by mediating the nuclear import of BRCA1 proteins in cancer cells (Alshareeda et al., 2015). Overexpression of IPO7 has been indicated and implicated as a distinguishing marker in prostate and lung cancer (Szczyrba et al., 2013; Lee et al., 2017). However, there is limited information about the biological function of IPO7 in carcinogenesis and its potential cargo proteins.

In the present study, we identified that IPO7 expression in CC was significantly upregulated compared with that in normal cervical tissues and associated with poor prognosis. We also explored the oncogenic function of IPO7 in CC cell proliferation *in vitro* and *in vivo*. Moreover, quantitative proteome profiling was performed to investigate potential IPO7 cargo proteins and first identified 32 novel IPO7 cargo proteins by PRM analysis. Our present results demonstrate the oncogenic function of IPO7 which is activating PI3K/AKT-mTOR signaling by mediating the nuclear import of GTF2I. Our work also shows that the expression of IPO7 negatively correlates with CD8+ T cell infiltration *via* regulating CD276 in CC which is a key immune checkpoint in tumor immunotherapy.

## Materials and Methods

### Clinical Specimens

Human cervical cancer patient tissues microarray obtained 15 normal cervix specimens, 72 cervical intraepithelial neoplasia specimens, and 94 cervical cancer specimens from Shanghai Jiao Tong University Affiliated Sixth People’s Hospital, with informed consent from all patients. All tissue specimens were confirmed by pathologist diagnosis.

### Cell Culture

Cell lines HeLa and C-4 I were preserved in Shanghai Cancer Institute, Ren Ji Hospital, School of Medicine, Shanghai Jiao Tong University and cultured in DMEM medium (GIBCO) with 10% FBS and (v/v) penicillin/streptomycin at 37°C in a humidified atmosphere with 5% CO_2_.

### Immunohistochemical Staining

Immunohistochemistry staining and scoring were performed according to previous research (Yang et al., 2021b). The primary antibody used was anti-IPO7 (dilution 1:1000, ab99273, Abcam). Antibodies against Ki67 (27309-1-AP) and anti-PCNA (10205-2-AP) were purchased from Proteintech (Chicago, United States).

### Quantitative Real-Time PCR

Total mRNA was extracted from cells using Trizol reagent (Takara) following the instructions. Quantitative real-time PCR (qRT-PCR) was performed with SYBR Green Super-mix (Takara) on ABI PRISM7500 Sequence Detection System (Applied Biosystems). Reference gene 18S was utilized to normalization. Primer sets used for IPO7 and 18s RNA examination were as follows: IPO7 forward 5′- CCCCAACACCATTATCGAGGC -3′, IPO7 reverse 5′- AGAGACTTGTGTGCTTCATTGAG -3′; 18s forward 5′-TGCGAGTACTCAACACCAACA-3′,18s reverse 5′- GCATATCTTCGGCCCACA-3′; B7H3 forward: 5′-ACAGGGC AGCCTATGACATT-3′, B7H3 reverse: 5′-GTCCTCAGCTCCT GCATTCT-3′

The formula RQ = 2−ΔCt was used to quantify gene expression levels for statistical analysis.

### RNA Interference

shRNAs against IPO7 were purchased from Gene Pharma (Shanghai, China). For IPO7 shRNA: shIPO7-1:5′- GATCCGC ATTCATCACATCATCAAACTTCAAGAGAGTTTGATGATGT GATGAATGCTTTTTTG3′; shIPO7-2:5′-GATCCGAACAGGG ATGTACCTAATGATTCAAGAGATCATTAGGTACATCCCTG TTCTTTTTTG -3′. siRNAs against GFT2I were also purchased from Gene Pharma (Shanghai, China). Transfection according to the manufacture’s protocols, using Lipofectamine 3000. For GFT2I siRNA: siGTF2I-1: 5- GCCAGAAUCACUAAAUUA -3, siGTF2I-2: 5- CAGCCACAGAAGAAAUAA -3.

### Western Blotting

Whole-cell lysates and separate nuclear/cytoplasmic fractions were extracted according to instructions and western blotting was performed as previously described (Yang et al., 2021b). Antibodies against GAPDH (60004-1-Ig), p-mTOR (67778-1-Ig), mTOR (20657-1-AP), p-AKT (66444-1-Ig), AKT (10176-2-AP), p-P70S6K (28983-1-AP), P70S6K (14485-1-AP), and GTF2I (10499-1-AP) were purchased from Proteintech (Chicago, United States). The antibodies anti-IPO7 (ab99273, Abcam).

### CCK-8, Colony, and Edu Assay

CCK-8 cell proliferation, Colony formation, and Edu stain assay were performed as previously described (Yang et al., 2021b).

### Liquid Chromatography–Mass Spectrometry

The tryptic peptides were dissolved in 0.1% formic acid (solvent A), and then separated using the EASY-nLC 1000 ultra-high performance liquid system. The peptides are separated by the ultra-high performance liquid system and injected into the NSI ion source for ionization and then analyzed by Orbitrap Fusion mass spectrometry. The ion source voltage is set to 2.2 kV, and the peptide precursor ions and their secondary fragments are detected and analyzed by high-resolution Orbitrap. The scanning range of the primary mass spectrum is set to 350–1550 m/z, and the scanning resolution is set to 60,000; the scanning range of the secondary mass spectrum is set to a fixed starting point of 100 m/z, and the secondary scanning resolution is set to 30,000. The resulting MS/MS data were processed using Maxquant search engine (v.1.5.2.8). Set the restriction enzyme digestion method to Trypsin/P; set the number of missed cleavage sites to 2; set the minimum peptide length to 7 amino acid residues. FDR was adjusted to <1% and minimum score for modified peptides was set >40.

### Parallel Reaction Monitoring Analysis

The peptides are from the remaining peptides of proteomics. The peptides are separated by the ultra-high performance liquid system and injected into the NSI ion source for ionization and then analyzed by the Q Exactive TM Plus mass spectrometer. The ion source voltage is set to 2.2 kV, and the peptide precursor ions and their secondary fragments are detected and analyzed by high-resolution Orbitrap. Peptide parameters: The protease is set to Trypsin (KR/P), and the maximum number of missed sites is set to 0. The peptide length is set to 7–25 amino acid residues, and cysteine alkylation is set as a fixed modification.

### Bioinformatic Analysis

We used the ‘‘Expression analysis-Box Plots’’ module of the GEPIA2 web server^1^ to obtain box plots of the expression difference between these tumor tissues and the corresponding normal tissues of the GTEx (Genotype-Tissue Expression) database and “Match TCGA normal and GTEx data” (Tang et al., 2019). The UALCAN portal^2^, an interactive web resource for analyzing cancer Omics data, allowed us to conduct protein expression analysis of the CPTAC (Clinical proteomic tumor analysis consortium) dataset (Chen et al., 2019). We used the ‘‘Immune-Gene’’ module of the TIMER2 web server and Sanger box^3^ to explore the association between IPO7 expression and immune infiltrates in cervical cancer. Meanwhile, we used the TISIDB database^4^ and verified the above results (Ru et al., 2019).

### Statistical Analysis

The SPSS 19.0 and GraphPad Prism 8.0 software was employed for statistical analysis. The student *t*-test was employed to analyze two groups of data. The one-way ANOVA was used for comprising of multiple groups. Error bars represent mean ± standard deviation (SD). Values of *P* < 0.05 were considered statistically significant.

## Results

### Overexpression of Importin 7 Promotes the Malignant Transformation of Cervical Cancer

**FIGURE 1:**
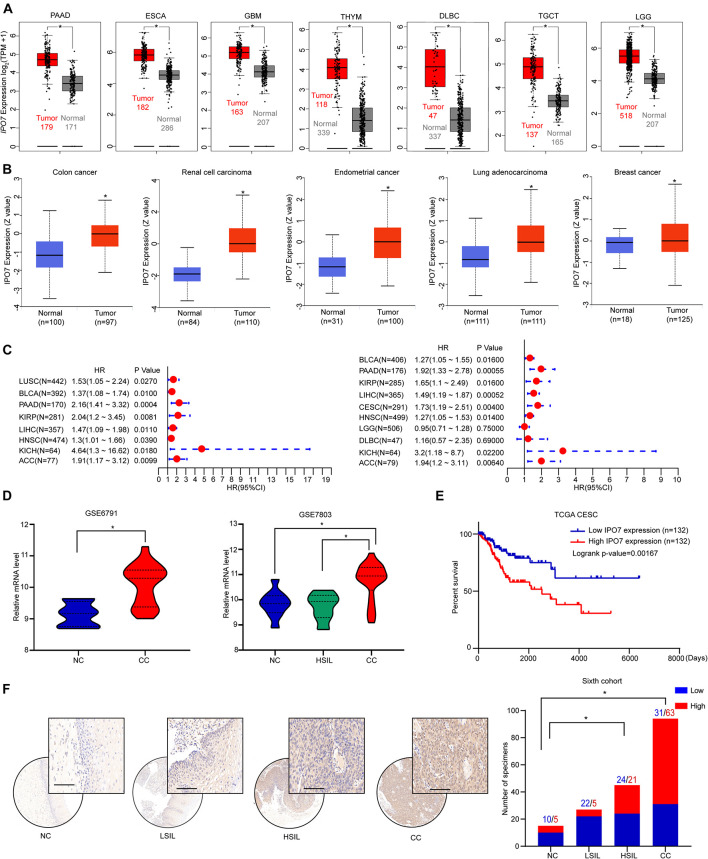
IPO7 is up-regulated and associated with a poorer prognosis in CC. **(A)** The expression of *IPO7* in the TCGA dataset compared to corresponding normal tissues of the GTEx dataset. **(B)** The protein level of IPO7 in primary tumor tissues and normal tissues from CPTAC datasets. **(C)** Correlation between IPO7 expression and overall survival and disease-free survival of cancers in the TCGA dataset. **(D)** Analysis of *IPO7* expression profiles in cervical cancer (CC), high-grade squamous intraepithelial lesion (HSIL), and normal cervix (NC) specimens from GSE6791 and GSE7803 datasets. **(E)** Kaplan-Meier analysis of the overall survival of patients with IPO7 high or low expression level. **(F)** Representative immunohistochemical images and quantification analysis showing IPO7 expression in CC, CIN, and CC specimens. LSIL is ligh-grade squamous intraepithelial lesion. Scale bar: 20 μm. The *P*-value was calculated by χ2 test or Fisher’s exact test. *p < 0.05.

To explore the potential oncogenic role of IPO7, we first applied the TCGA and GTEx datasets to analyze and results showed that the gene expression of *IPO7* was significantly increased in the various cancers (Figure 1A). The CPTAC dataset also showed a higher level of IPO7 protein in the various primary tumor tissues (Figure 1B). Meanwhile, high expression of IPO7 was correlated with of poorer prognosis for cancer patients (Figure 1C). To further evaluate the expression of IPO7 in cervical cancer (CC), we initially investigated the gene expression profiles from the GSE6791 and GSE7803 datasets. These results show that *IPO7* expression was significantly increased in CC compared to normal cervix tissues (Figure 1D). Moreover, higher expression of *IPO7* was well correlated with a poorer prognosis of CC patients (Figure 1E). Furthermore, the immunohistochemistry staining assay in a tissue microarray (TMA) validated that IPO7 was primarily located in the nucleus of CC tissues and that the protein level of IPO7 was significantly higher in CC tissues than in the normal cervix and CIN (cervical intraepithelial neoplasia) tissues (Figure 1F). Therefore, we speculate that IPO7 has potential as an oncogenic biomarker for the progression of CC.

### Knockdown of Importin 7 Inhibits Cervical Cancer Cell Proliferation *in vitro* and *in vivo*

**FIGURE 2:**
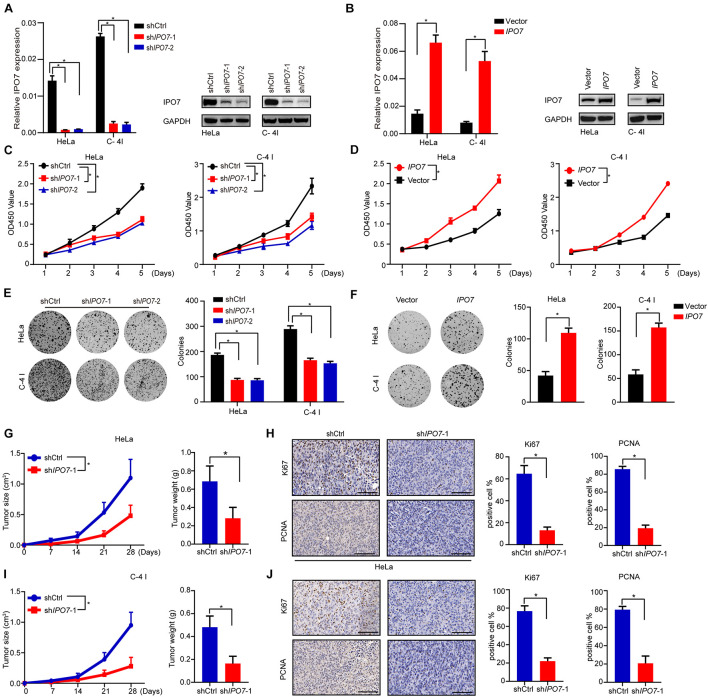
IPO7 promotes the cell proliferation of CC *in vitro*. **(A,B)** The expression level in Hela and C-4 I after IPO7 knockdown or overexpression. **(C,D)** CCK-8 assay analyzes cell viability after IPO7 knockdown or overexpression. **(E,F)** Representative colony formation and quantification analysis. **(G–J)** Hela and C-4 I cells were infected with shIPO7 or shCtrl and injected into nude mice. Representative time course of xenograft growth and tumor weight **(G,I)**. Representative typical images of PCNA and Ki67 and quantification analysis from shCtrl and shIPO7 groups **(H,J)**. Error bars represent mean ± standard deviation (SD). Scar bar: 50 μm. Two-tailed *t*-test, *p < 0.05.

To investigate the biological functions of IPO7 in the proliferation of CC cells, HeLa, and C-4I, two CC cell lines with relatively higher expression levels of IPO7, were selected (Supplementary Figures 1A,B). The efficiency of IPO7 knockdown and overexpression was confirmed by quantitative real-time PCR and immunoblot analyses, respectively (Figures 2A,B). Compared to shCtrl cells, HeLa, and C-4I cells with IPO7 interference had significantly reduced cell viability (Figure 2C). Conversely, the overexpression of IPO7 significantly increased cell viability (Figure 2D). To further analyze the role of IPO7 in CC cell proliferation, we performed a clonogenicity assay and verified the above results (Figures 2E,F). To further verify the IPO7 oncogenic function in CC cell proliferation, a subcutaneous xenograft model was performed by injecting with sh*IPO7* in HeLa and C-4I cells. The CC tumor growth in the sh*IPO7* groups was remarkably inhibited, as evaluated by tumor weight and volume measurements (Figures 2G,I). Furthermore, IHC staining results revealed that the immunostaining intensities of PCNA and Ki-67, markers of cell proliferation, were significantly decreased in the sh*IPO7* groups (Figures 2H,J). As expected, overexpression of IPO7 promoted the tumor growth (Supplementary Figures 2A–D). Taken together, these results suggested that IPO7 was profoundly implicated in promoting CC proliferation.

### Functional and Network Analyses of Cargo Proteins Recognized by Importin 7

**FIGURE 3:**
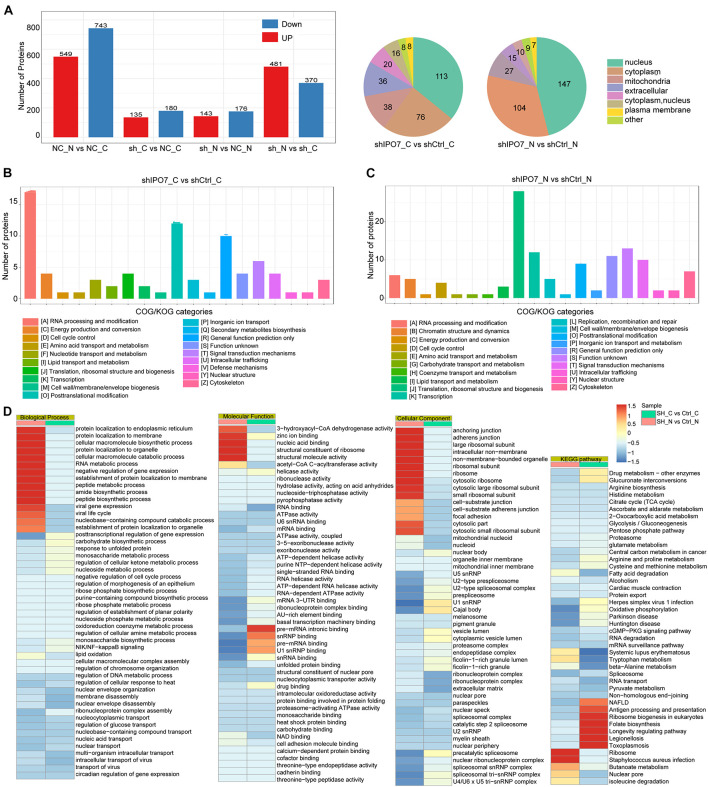
Comparative profiling of the proteomes after IPO7 knockdown. **(A)** Results showing protein expression variations in the nucleus and cytoplasm after IPO7 knockdown, compared with shCtrl groups, *n* = 3. **(B,C)** Differentially expressed proteins of the cytoplasm **(B)** and nucleus **(C)** after IPO7 knockdown were analyzed with Clusters of Orthologous Groups of proteins (COG/KOG) database. **(D)** The significantly enriched biological process, cellular component, molecular function, and Kyoto Encyclopedia of Genes and Genomes (KEGG) pathway terms were shown.

Because IPO7, as a member of Kapβs, regulated cell functions mainly by transporting cargo proteins into the nucleus, the liquid chromatography–mass spectrometry (LC-MS/MS) analysis was performed to explore IPO7 cargo proteins. Expression alterations in the separate nuclear/cytosolic proteomes were analyzed upon IPO7 knockdown in HeLa cells (Supplementary Table 1). Comparative proteome analysis was conducted on three samples per group, and these differentially expressed proteins were identified with >1.5-fold changes and *p*-values <0.05. Comparative proteome profiling revealed hundreds of differentially expressed proteins in the shIPO7 group compared to shCtrl group (Figure 3A) and mainly concentrated in in the nucleus and cytoplasm (Figure 3B).

To fully understand the significant biological functions and pathways associated with IPO7, these differential proteins in the nucleus or cytoplasm were investigated based on bioinformatic analysis. COG/KOG categories analysis results showed that these differential proteins of cytoplasm and nucleus mainly were enriched in RNA processing and post-translational modification (Figure 3C). Further the top enriched GO and KEGG pathway terms are shown in Figure 3D. Biological process category analysis demonstrated that these proteins with enhanced localization in the cytoplasm were linked with post-translational regulation of proteins transport and RNA metabolic processes. Those proteins with reduced localization in the nucleus mainly were enriched in nucleic acid transport and gene expression. Molecular function category analysis showed that these differential proteins of cytoplasm were involved in nucleic acid binding and mRNA processing. Those differential proteins of nucleus mainly were enriched in RNA and DNA binding activity. The cellular component category indicated that these differential proteins of cytoplasm and nucleus were enriched in ribosome and organelle part. The KEGG pathway analysis revealed that the differentially expressed proteins mainly participated in RNA transport and spliceosome-associated pathways.

**FIGURE 4:**
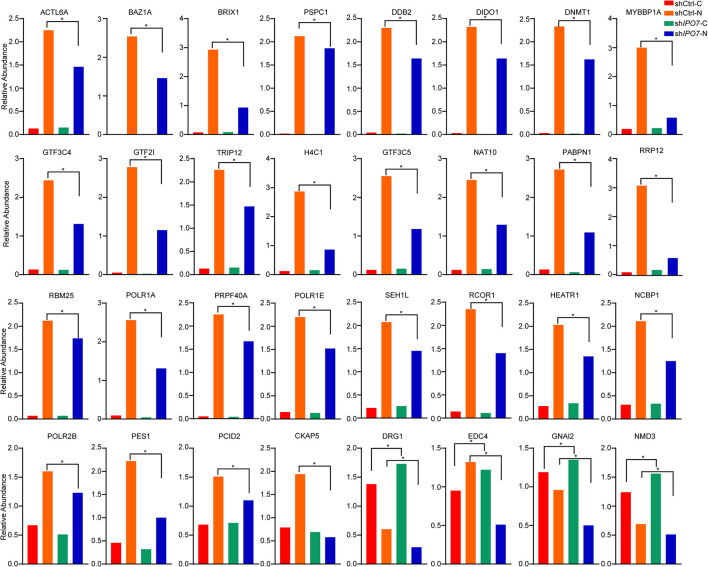
PRM analyses of the differentially expressed proteins. Parallel reaction monitoring (PRM) analysis of the differentially expressed proteins after IPO7 knockdown relative to the shCtrl groups. ACTL6A, BAZ1A, BRIX1, PSPC1, DDB2, DIDO1, DNMT1, MYBBP1A, GTF3C4, GTF2I, TRIP12, H4C1, GTF3C5, NAT10, PABPN1, RRP12, RBM25, POLR1A, PRPF40A, CHD4, POLR1E, SEH1L, RCOR1, HEATR1, NCBP1, POLR2B, PES1, PCID2, CKAP5, DRG1, EDC4, GNAI2, and NMD3 were detected. Two-tailed Student’s *t*-test, *p < 0.05.

To further verify the nuclear/cytosolic localization of different proteins, parallel reaction monitoring (PRM) was utilized for label-free quantification by a MS. Limited by the characteristics of some proteins and the abundance of their expression, 32 potential cargo proteins were further independently validated by PRM analysis (Supplementary Table 2). Each protein was quantified with more than two unique peptides. Some proteins only identified one peptide due to sensitivity and other reasons. Overall, these PRM results mirrored the results from the nuclear/cytosolic proteome analysis-based experiment. Similar to other proteomics studies, there were some discrepancies between the two quantitative datasets (Xu et al., 2012; Wu et al., 2019). As shown in Figure 4, DNA-templated transcription-associated proteins, such as DIDO1, DNMT1, and RCOR1, mainly accumulated in the nucleus and knockdown of IPO7 significantly reduced their nuclear localization. The nuclear localization of transcription regulation-associated proteins, including PPABPN1, NCBP1, THOC3, GTF3C4, and GTF3C5, in the IPO7-knockdown group was reduced. Moreover, there were some cell division- and nucleoprotein complex localization-associated proteins, such as CKAP5, GNAI2, NMD3, and PCID2, representing the scattered localization in the nucleus and cytoplasm. PRM analysis results showed that their nuclear localization was dramatically decreased and redistributed to the cytoplasm when IPO7 was knocked down.

### Knockdown of Importin 7 Inhibited PI3K/AKT-mTOR Signaling Pathway in Cervical Cancer Cell

**FIGURE 5:**
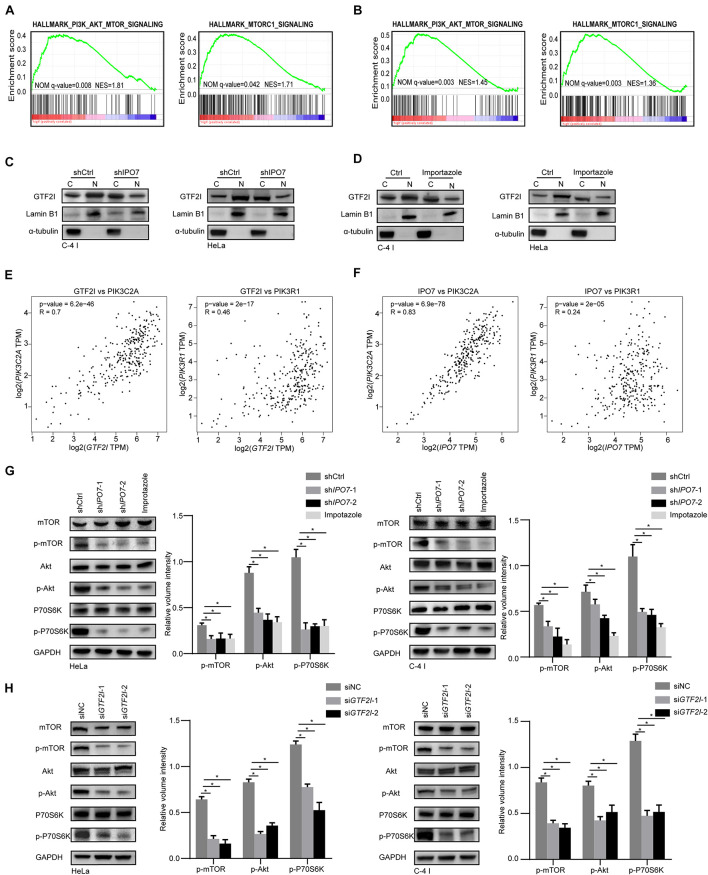
IPO7 regulates the activation of PI3K/AKT-mTOR pathway. **(A,B)** Gene set enrichment analysis (GSEA) shows the enriched gene expression signature of IPO7 and GTF2I in the TCGA datasets. **(C,D)** Immunoblots analysis shows the GTF2I levels in the nucleoplasm and cytoplasm after transfecting with shIPO7 or Importazole. **(E)** The correlation of GTF2I with PIK3CA and PIK3C2A. **(F)** The correlation of IPO7 with PIK3CA and PIK3C2A. **(G)** Immunoblot analysis of phosphor-mTOR (p-mTOR), mTOR, phosphor-AKT(p-AKT), AKT, phosphor-P70S6K (p-P70S6K), P70S6K expression in shCtrl, sh*IPO7*, and Importazole groups. The relative expressions were quantified by normalizing to GAPDH. **(H)** Immunoblot analysis of phosphor-mTOR (p-mTOR), mTOR, phosphor-AKT(p-AKT), AKT, phosphor-P70S6K (p-P70S6K), P70S6K expression in siNC and si*GTF2I* groups. The relative expressions were quantified by normalizing to GAPDH. Error bars represent mean ± standard deviation (SD). *p < 0.05.

To further elucidate the molecular machinery correlation with IPO7 oncogenic function, we compared gene expression profiles of IPO7 high expression patients to low expression patients from TCGA datasets. Gene set enrichment analysis (GSEA) showed that the PI3K-AKT signaling pathway and mTORC1 pathway were significantly altered (Figure 5A). Previous studies demonstrated that karyopherin-mediated cargo proteins may play an important role in promoting cancer progression (Yang et al., 2021a). Then, we found that only the high expression of GTF2I and TRIP12 was associated with the poor prognosis of CC patients in 32 different proteins (Supplementary Figures 3A,B). Meanwhile, GSEA indicated that the high gene expression profiles of GTF2I were also enriched in the PI3K/AKT-mTOR signaling pathway (Figure 5B). Immunoblot analysis of separate nuclear/cytoplasmic fractions showed that the nuclear localization of GTF2I was decreased when IPO7 was knocked down (Figure 5C). Moreover, Importazole, an inhibitor of IPO7, decreased the nuclear localization of GTF2I (Figure 5D). GTF2I, as a regulator of transcription, may associates with the expression of some key genes involving in the PI3K-AKT signaling pathway. Indeed, a positive correlation of GTF2I with PIK3R1 and PIK3C2A was detected in the TCGA CECS database (Figure 5E). Moreover, we could detect a positive correlation of IPO7 with PIK3R1 and PIK3C2A, which indicated that IPO7 may contribute to regulating the PI3K/AKT-mTOR signaling pathway by mediating the nuclear import of GTF2I (Figure 5F). We further performed a western blot assay to examine the activation of PI3K/AKT signaling in sh*IPO7* HeLa and C-4 I cells and knockdown of IPO7 or Importazole were significantly inhibited the activation of AKT and its downstream targets, mTOR and P70S6K (Figure 5G). Moreover, immunoblot analysis showed that the activation of PI3K/Akt-mTOR was inhibited when GTF2I was knocked down in HeLa and C-4 I cells (Figure 5H). Altogether, these results demonstrated that IPO7 mediated the nuclear import of GTF2I and contributed to activating PI3K/AKT-mTOR signaling pathway.

### The Importin 7 Negatively Associates With CD8 T Cell Infiltration in Cervical Cancer

**FIGURE 6:**
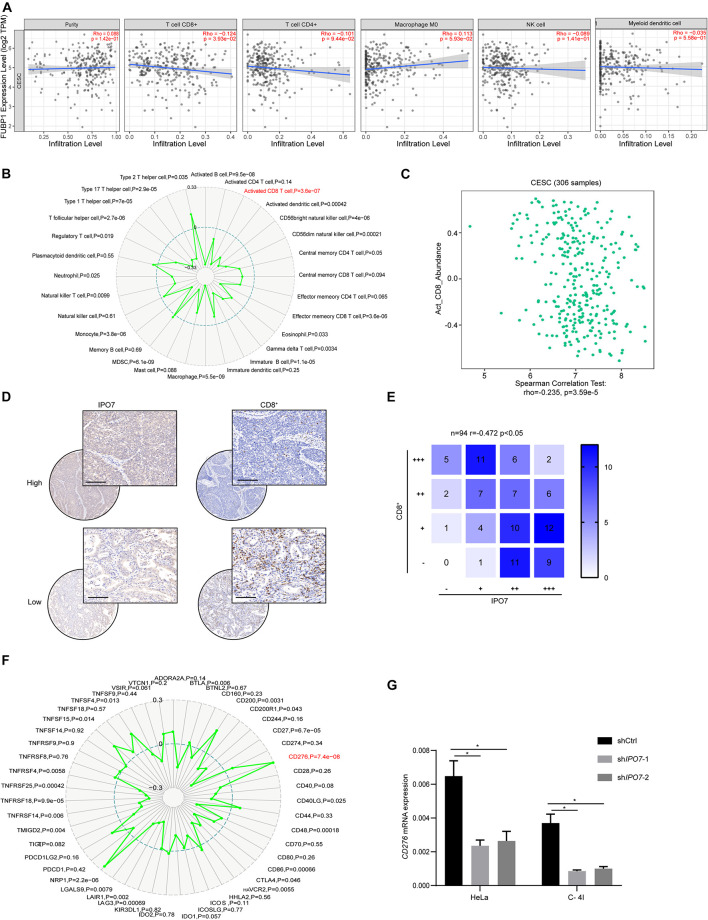
The IPO7 negatively correlates with CD8 T cell infiltration in cervical cancer. **(A)** The correlation between IPO7 with immune cell type infiltrates are analyzed by CIBERSORT algorithm. **(B)** Gene-immune analysis of IPO7 in CC conducted on Sanger box. **(C)** The correlation between IPO7 with activated CD8 cell infiltrate level is analyzed by TISIDB database. **(D)** Representative IHC images of IPO7 and CD8^+^ cells in CC tissues. Upper panel is IPO7 with high expression; lower panel is IPO7 with low expression in CC. Scale bar, 50 μm. **(E)** Correlation analysis of IPO7 and CD8^+^ cells in CC. **(F)** Gene-immune checkpoints analysis of IPO7 in CC conducted on Sanger box. **(G)** The mRNA level of CD276 in Hela and C-4 I cells under knockdown of IPO7. Error bars represent mean ± standard deviation (SD). Two-tailed *t*-test, *p < 0.05.

Immune infiltration in the tumor microenvironment (TME) plays a key role in tumor progression and the efficacy of immune checkpoint immunotherapies. To further explore the impact of IPO7 in the tumor immune microenvironment, we first quantified the correlation of IPO7 and the infiltration levels of the immune cell types in CC samples by the TIMER database. As shown in Figure 6A, a negative correlation of IPO7 with CD8 T cell infiltration was detected. We found the expression of IPO7 had the strongest negative correlation with activated CD8 T cell by using Gene-Immune Analysis using Sanger box^3^ (Figure 6B). Consistently, we also performed a TISIDB database and verified the above results (Figure 6C). Furthermore, the immunohistochemistry staining assay in a TMA validated that the expression of IPO7 inversely correlated with CD8 T cell infiltration in CC tissues (Figures 6D,E). Numerous studies have demonstrated that immune checkpoints on cancer cell surfaces participated in tumor immune evasion. Therefore, we analyzed that the correlation of IPO7 and the expression of the immune checkpoint gene by the TIMER database. As shown in Figure 6F, a strong positive correlation of IPO7 with expression of CD276 was detected. Moreover, knockdown of IPO7 remarkedly suppressed CD276 expression in HeLa and C-4 I cells (Figure 6G). In this present study, we found that IPO7-related cargo proteins mainly were enriched in gene transcription and post-translational modification. Therefore, we measured the mRNA levels of IPO7, cargo proteins, and CD276 in the CESC TCGA database and analyzed their correlations. A positive correlation of IPO7 and cargo proteins with the expression of CD276 was detected in CC patients (Supplementary Figure 4A). Taken together, IPO7 may contribute to regulating the tumor immune microenvironment of CC.

## Discussion

Importin 7 is an essential nuclear import receptor of oncogenes and tumor suppressor genes. As a member of the importins, aberrant expression of the *IPO7* gene has been recently reported in a multitude of malignancies and is correlated with tumorigenesis and aggressiveness, and IPO7 has evolved as a potential cancer therapeutic intervention target. This study intended to elucidate the molecular mechanisms and explore potential cargo proteins.

Malfunction of the nuclear transport machinery leads to abnormal spatiotemporal expression of cargo proteins and may allow these genes to become novel biomarkers and therapeutic targets. The dysregulation of karyopherin is frequently observed to be involved in multiple tumors. For example, overexpression of KPNA2 is linked with poor survival in bladder cancer (Zeng et al., 2021). In this study, widespread computational bioinformatics analyses of the GEO database and clinical TAM analysis results showed that IPO7 expression was remarkably upregulated and worsened the prognosis of CC patients. Moreover, our work indicated that perturbed expression of *IPO7* affected the proliferation of CC cells. Therefore, this report reveals that *IPO7* may have an oncogenic function in CC progression.

It has been well established that IPO7 is involved in regulating cell functions mainly by transporting special cargo proteins into the nucleus. For example, IPO7 regulates the nuclear import of FOXO3 in a redox-sensitive and disulfide-dependent manner (Putker et al., 2015). The oncogenic function of karyopherin is also attributed to the disordered nuclear-cytoplasmic transport of cargo proteins (Wang et al., 2012; Vuorinen et al., 2017). Previous studies have indicated that IPO7 promoted glioblastoma cell proliferation and migration by increasing the nuclear import of GLI1 (Xue et al., 2015). To reveal the potential cargo proteins of IPO7, herein, the proteomes were comparatively profiled using LC-MS/MS quantitative analysis. Compared with the shCtrl group, the experimental group revealed a large number of differential proteins in the nucleus and cytoplasm. The bioinformatics analysis proposed that these differential proteins were significantly enriched for the gene expression and post-translational modification. A previous study also indicated that the karyopherin IPO7 is involved in RNA processes or gene transcription (Twyffels et al., 2014).

In the PRM analysis, 32 potential cargo proteins were primarily related to the transcription and mRNA splicing pathways. Previously, these cargo proteins and associated pathways have been preliminarily implicated in tumorigenesis and aggressiveness. For example, PSPC1 activates metastatic reprogramming by increasing TGF-β1 secretion and promotes EMT in cancer cells (Yeh et al., 2018). As a splicing factor, RBM25 contributes to regulating MYC activity in acute myeloid leukemia (Ge et al., 2019). GTF2I, PES1, and RCOR1 were involved in RNA and DNA binding pathways. PES1 is upregulated and improves hepatocellular carcinoma cells proliferation (Wang et al., 2019). GTF2I affected the expression of critical activators, such as PIK3R1, in PI3K signaling pathway by regulating a highly conserved DNA element in embryonic fibroblasts (Segura-Puimedon et al., 2013). In the present study, we found that IPO7 activates PI3K/AKT-mTOR pathway to exert oncogenic function in CC cells by transporting GTF2I into the nucleus, which could provide clear information for the future of tumor treatment research.

In conclusion, the current study demonstrates that IPO7 was upregulated and closely correlated with a poor prognosis in CC. Further quantitative proteomics and PRM analyses demonstrated that IPO7 cargo proteins were mainly involved in oncogene transcription and mRNA splicing pathways. Intriguingly, in addition to its carcinogenic functions, our work also indicated that IPO7 negatively correlated with CD8 T cell infiltration in CC. These findings strongly expand the understanding of the IPO7 cargo protein and might act as a novel therapeutic strategy for cancer treatment.

## Data Availability Statement

The datasets presented in this study can be found in online repositories. The names of the repository/repositories and accession number(s) can be found below: PXD026995, http://proteomecentral.proteomexchange.org/cgi/GetDataset?ID=PXD026995.

## Ethics Statement

The animal study was reviewed and approved by Shanghai Sixth People’s Hospital Animal Care and Use Committee. Written informed consent was obtained from the individual(s) for the publication of any potentially identifiable images or data included in this manuscript.

## Author Contributions

BY and YT designed the research and wrote the manuscript. JC and YH performed the experiments. YH performed the statistical analysis. All authors contributed to manuscript revision, read, and approved the submitted version.

## Conflict of Interest

The authors declare that the research was conducted in the absence of any commercial or financial relationships that could be construed as a potential conflict of interest.

## Publisher’s Note

All claims expressed in this article are solely those of the authors and do not necessarily represent those of their affiliated organizations, or those of the publisher, the editors and the reviewers. Any product that may be evaluated in this article, or claim that may be made by its manufacturer, is not guaranteed or endorsed by the publisher.

## Footnotes

1
http://gepia2.cancer-pku.cn/#analysis

2
http://ualcan.path.uab.edu/analysis-prot.html

3
http://sangerbox.com/Index

4
http://cis.hku.hk/TISIDB/
