# Supplementary material for: Increased Nuclear Transporter Importin 7 Contributes to the Tumor Growth and Correlates With CD8 T Cell Infiltration in Cervical Cancer
Source: Front Cell Dev Biol. 2021 Sep 28;9:732786. doi: 10.3389/fcell.2021.732786 (PMC8505702; doi:10.3389/fcell.2021.732786)
Supplement: Supplementary file 1 [file Data_Sheet_1.PDF]

Table 1 Basic statistics analysis of Mass Spectrometry data results

Total spectrum	Matched spectrum	Peptides	Unique peptides	Identified proteins	Quantifiable proteins
464159.0	285714	48409.0	46205.0	4884.0	3690.0

Table 2 PRM protein peptide quantification results

				Normalized Area			
Protein Accession	Protein Gene	Peptide	Retention Time	Cytoplasm (n=3)		Nucleus (n=3)	
				shCtrl	shIPO7	shCtrl	shIPO7
O00541	PES1	LQLSLADFR	33.68	0.44	0.22	2.35	0.99
O00541	PES1	YPTFIDALR	32.91	0.47	0.43	2.10	1.00
P26358	DNMT1	NQLCDLETK	10.67	0.00	0.00	2.04	1.96
P26358	DNMT1	LPLFPEPLHVFAPR	42.52	0.06	0.04	2.62	1.27
Q09161	NCBP1	IFANTESYLK	20.39	0.33	0.21	2.22	1.23
Q09161	NCBP1	ATNDEIFSILK	35.14	0.29	0.45	2.01	1.26
Q14669	TRIP12	VEPVGNAPLLALVHK	33.83	0.15	0.22	2.17	1.45
Q14669	TRIP12	TCPFFFPFDTR	43.34	0.10	0.07	2.35	1.48
Q92466	DDB2	YNLIVVGR	22.86	0.03	0.02	2.41	1.54
Q92466	DDB2	TIDVFDGNSGK	16.32	0.04	0.03	2.19	1.74
Q9H0A0	NAT10	ESLQDTQPVGVLVDCCK	26.56	0.17	0.17	2.26	1.40
Q9H0A0	NAT10	IVSGCPLPEACELYYVNR	33	0.08	0.11	2.63	1.18
Q9NRL2	BAZ1A	LHILASGADVTSANAK	15.97	0.00	0.00	2.74	1.26
Q9NRL2	BAZ1A	LSSTSVYDLTPGEK	20.62	0.00	0.00	2.35	1.65
O75400	PRPF40A	TPAEQLLSK	13.49	0.04	0.02	2.16	1.78
O75400	PRPF40A	EPAFEDITLESER	27.08	0.06	0.05	2.33	1.56
O95602	POLR1A	SLPCFEPYEFTPR	35.74	0.11	0.08	2.61	1.19
O95602	POLR1A	YAEVLDLR	21.7	0.08	0.00	2.50	1.42
O96019	ACTL6A	TAVLTAFANGR	21.08	0.09	0.09	2.45	1.38
O96019	ACTL6A	IPEGLFDPSNVK	27.88	0.17	0.22	2.06	1.54
P04899	GNAI2	IAQSDYIPTQQDVLR	24.79	1.08	1.46	0.95	0.51
P04899	GNAI2	EIYTHFTCATDTK	13.88	1.30	1.24	0.97	0.49
P30876	POLR2B	VSGDDVIIGK	13.44	0.67	0.51	1.60	1.23
Q14008	CKAP5	NLGIPIITVLGDSK	45.24	0.70	0.63	2.04	0.63
Q14008	CKAP5	VNDFLAEIFK	44.19	0.89	0.74	1.84	0.53
Q14839	CHD4	EFSTNNPFK	16.81	0.02	0.02	2.21	1.76
Q14839	CHD4	YAILNEPFK	25.66	0.00	0.00	2.36	1.64
Q5JTH9	RRP12	SWLLPVIR	39.66	0.11	0.19	3.10	0.60
Q5JVF3	PCID2	DGASCAELVSFK	22.01	0.82	0.72	1.40	1.06
Q5JVF3	PCID2	VFANNADQQLVK	14.8	0.54	0.70	1.62	1.14
Q6P2E9	EDC4	LCTQLEGLQSTVTGHVER	24.92	0.95	1.22	1.32	0.51
Q8WXF1	PSPC1	YGEPSEVFINR	20.42	0.02	0.01	2.12	1.86
Q96EE3	SEH1L	TAAVWEEIVGESNDK	32.01	0.19	0.37	2.13	1.32
Q96EE3	SEH1L	VQIFEYNENTR	20.15	0.24	0.15	2.02	1.59
Q96J01	THOC3	TASVFLLEK	25.17	0.14	0.07	2.22	1.56
Q9BQG0	MYBBP1A	SPAESCDLLGDIQTCIR	34.12	0.11	0.15	2.97	0.77

Q9BQG0	MYBBP1A	VLDLVEVLVTK	44.12	0.28	0.29	3.03	0.40
Q9GZS1	POLR1E	LSYVGNNFGTGALK	24.21	0.15	0.13	2.20	1.52
Q9H583	HEATR1	LALPQSDASLLSR	28.48	0.28	0.34	2.03	1.35
Q9UKL0	RCOR1	VGPQYQAVVPDFDPAK	30.95	0.14	0.11	2.35	1.40
Q9UKN8	GTF3C4	QVDLIDLVR	35.61	0.13	0.12	2.44	1.31
Q9Y295	DRG1	IQLLDLPGIIEGAK	46.55	1.45	1.84	0.53	0.18
Q9Y295	DRG1	SDATADDLIDVVEGNR	33.55	1.31	1.63	0.67	0.39
Q9Y5Q8	GTF3C5	EGYNNPPISGENLIGLSR	31.69	0.13	0.20	2.67	1.00
Q9Y5Q8	GTF3C5	IYQVLDFR	28.08	0.10	0.09	2.44	1.36
P49756	RBM25	FEDEDSDDVPR	9.8	0.07	0.07	2.12	1.74
P62805	H4C1	ISGLIYEETR	19.08	0.12	0.18	2.89	0.81
P62805	H4C1	VFLENVIR	27.39	0.12	0.12	2.85	0.91
P78347	GTF2I	SPSWYGIPR	22.57	0.04	0.02	2.76	1.19
P78347	GTF2I	EFSFEAWNAK	28.84	0.06	0.02	2.80	1.12
Q86U42	PABPN1	TSLALDESLFR	32.98	0.13	0.06	2.72	1.09
Q8TDN6	BRIX1	FLVQNIHTLAELK	30.85	0.07	0.08	2.93	0.93
Q96D46	NMD3	LISQDIHSNTYNYK	12.98	1.21	1.45	0.79	0.55
Q96D46	NMD3	VPDVVLIK	24.2	1.26	1.68	0.58	0.47
Q9BTC0	DIDO1	ICTGQVPSAEDEPAPK	13.14	0.05	0.00	2.32	1.63
Q9BTC0	DIDO1	AFDTQLVER	17.41	0.02	0.00	2.33	1.64
Q9ULW0	TPX2	DDINLLPSK	21.3	0.00	0.00	2.26	1.74
Q9ULW0	TPX2	STAELEAEELEK	15.8	0.07	0.00	2.34	1.58
