# Supplementary material for: Increased Nuclear Transporter Importin 7 Contributes to the Tumor Growth and Correlates With CD8 T Cell Infiltration in Cervical Cancer
Source: Front Cell Dev Biol. 2021 Sep 28;9:732786. doi: 10.3389/fcell.2021.732786 (PMC8505702; doi:10.3389/fcell.2021.732786)
Supplement: Supplementary file 2 [file Data_Sheet_2.PDF]

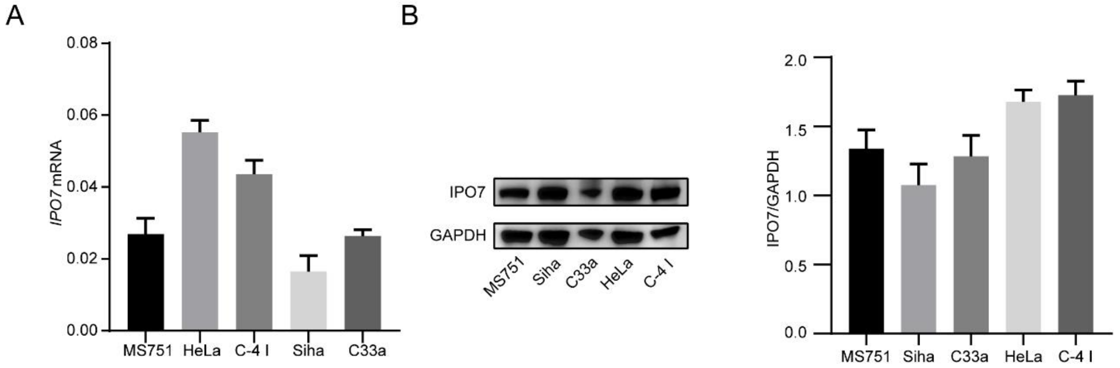

Supplementary Figure 1 (A) The expression of *IPO7* mRNA was detected by real-time quantitative PCR in CC cell lines. (B) The protein level of IPO7 was analyzed by western blotting, Error bars represent mean ± standard error of mean.

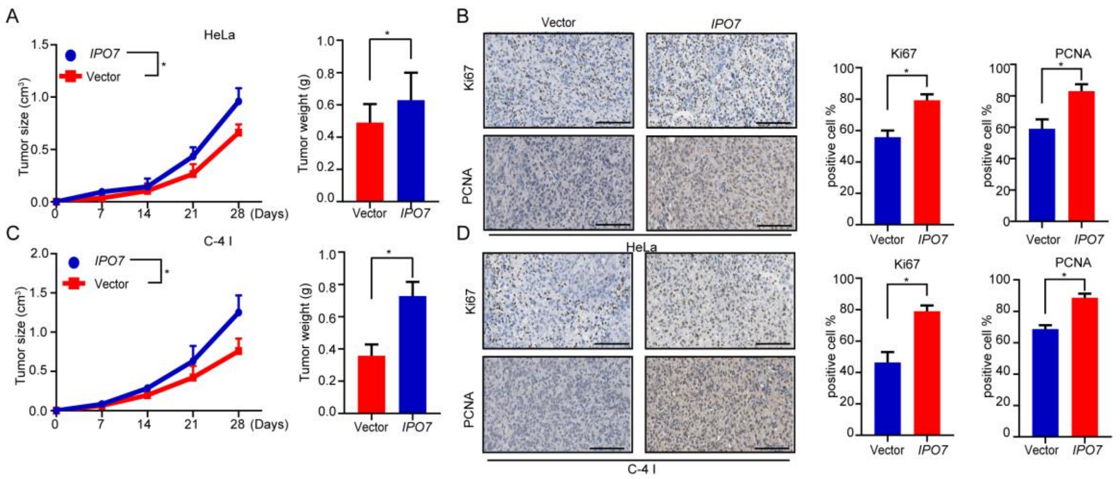

Supplementary Figure 2 (A-D) Hela and C-4 I cells were infected with vector or overexpression of IPO7 and injected into nude mice. Representative time course of xenograft growth and tumor weight (A, C). Representative typical images of PCNA and Ki67 and quantification analysis (B, D). Error bars represent mean ± standard deviation (s.d.). Scar bar: 50μm. Two-tailed t-test, *$p < 0.05$.

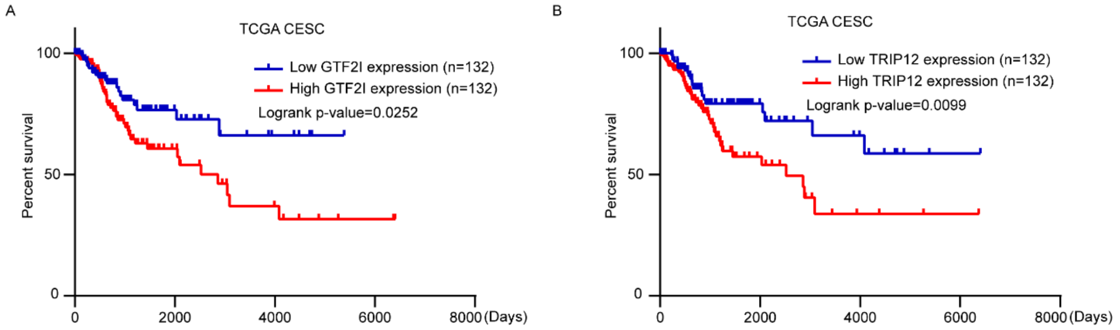

Supplementary Figure 3 (A, B) Kaplan-Meier analysis of the overall survival of patients with GTF2I and TRIP12 high or low expression level.

A

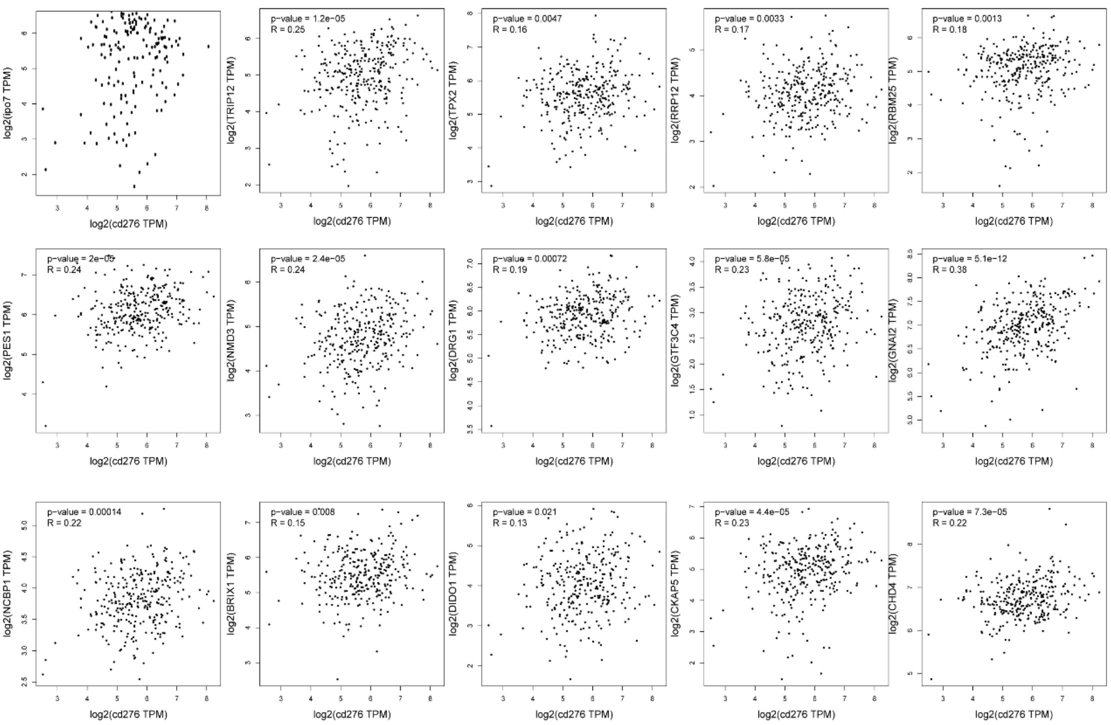

Supplementary Figure 4 (A) The correlation between the expression of IPO7, TRIP12, TPX2, RRP12, RBM25, PES1, MMD3, DRG1, GTF3C4, GNAI2, NCBP1, BRIX1, DIDO1, CKAP5, CHD4 and the expression of CD276 in CC samples were then analyzed and the results are shown.
